# Prevalence of Y chromosome microdeletions of the azoospermia factor region among Libyan men with infertility

**DOI:** 10.3892/mi.2026.325

**Published:** 2026-05-26

**Authors:** Sassia O. Regeai, Abdul Hakim S. Elnfati, Nuzha M. Alomrani, Afaf Shibane, Salem Ahlees, Osama Almajdoub, Ibrahim Ginaw, Mohamed Abdusalam, Ashraf Zaied, Inas M. Alhudiri, Adem I. Elzagheid

**Affiliations:** 1 Department of Histology and Genetics, Faculty of Medicine, University of Tripoli, Tripoli, Libya; 2 Yashfeen Clinic, Tripoli, Libya; 3 Department of Zoology, Faculty of Science, University of Tripoli, Tripoli, Libya; 4 Department of Genetic Engineering, Libyan Biotechnology Research Center, Tripoli, Libya; 5 Nour Al-Hayat Fertility Clinic, Tripoli, Libya; 6 Al Khalil Hospital, Tripoli, Libya

**Keywords:** Y chromosome microdeletions, azoospermia factor, non-obstructive azoospermia, hypergonadotropism, male infertility

## Abstract

The detection of Y chromosome microdeletions (YCMDs) in the azoospermia factor (AZF) region is a critical diagnostic tool for male infertility. The present study aimed to determine the prevalence of YCMDs among Libyan men with infertility following the European Academy of Andrology (EAA) and the European Molecular Genetics Quality Network (EMQN) guidelines. The present descriptive cross-sectional study included 41 men with infertility, stratified into two primary groups for comparative analysis: One group with azoospermia (n=23) and a non-azoospermia group (n=18). Genetic screening for AZFa, AZFb and AZFc microdeletions was performed using multiplex PCR with standardized sequence-tagged site primers (sY84, sY127, sY254, sY86, sY134 and sY255). Hormonal profiles, including follicle stimulating hormone (FSH), luteinizing hormone (LH), testosterone (T) and prolactin (PRL), as well as their ratios, were evaluated. The overall prevalence of YCMDs was 2.44% (1/41; 95% confidence interval, 0.06-12.79%). A single case of a complete AZFb+c microdeletion was identified in a patient with azoospermia. The azoospermia group exhibited significantly higher mean FSH (P<0.001) and LH levels (P<0.01) than those of the non-azoospermia group. The azoospermia group demonstrated a suppressed LH/FSH ratio (0.61) and a reduced T/LH ratio (0.37), indicating testicular failure and compensated Leydig cell dysfunction, respectively. To the best of our knowledge, the present study represents the first investigation in Tripoli, Libya, to report the prevalence of YCMDs in accordance with the EAA/EMQN international standards. Future large-scale, multicenter studies incorporating larger cohorts, fertile control groups, and broader geographic representations, are essential to establish a comprehensive genetic landscape of male infertility in Libya.

## Introduction

Male infertility is a considerable and steadily increasing global reproductive health burden (1,2). Numerical and structural chromosomal abnormalities represent one of the most critical genetic etiologies; ~5-15% of all men with infertility and up to 25% of those with severe spermatogenic failure (i.e., oligozoospermia or azoospermia) carry a chromosomal anomaly (3,4). G-banding cytogenetics remains the clinical gold standard for the initial genetic evaluation of these patients, as it is essential for identifying numerical anomalies (e.g., 47, XXY; 47, XYY) and large-scale structural rearrangements (>5-10 Mb) such as translocations and inversions. However, standard cytogenetic analyses are limited by its resolution (3,4).

Y chromosome microdeletions (YCMDs) are the most frequent structural abnormalities contributing to male infertility and are the second most common genetic cause of spermatogenic impairment following Klinefelter syndrome (5,6). As YCMDs typically range from 0.8 to 4.0 Mb, they are below the resolution limit of G-banding. Furthermore, the highly repetitive nature of Y chromosomal DNA lacks sufficient unique sequence required to microscopically visualize such minute deletions. Consequently, the Y chromosome may appear structurally normal under a microscope despite the presence of clinically relevant microdeletions. Therefore, sequence-tagged site PCR (STS-PCR) is the gold standard for YCMD detection, as it enables the identification of specific deleted genomic sequences. Nevertheless, cytogenetic analysis remains a crucial diagnostic and prognostic element that ensures a comprehensive evaluation to guide reproductive therapy (4).

The Y chromosome plays a pivotal role in male sex determination and fertility by harbouring genes vital for gonadal differentiation, germ cell development, and spermatogenesis. The azoospermia factor (AZF) locus on the long arm (Yq11) contains essential gene clusters required for sperm production (7). Deletions within this region can result in varying degrees of spermatogenic impairment. The AZF region is particularly susceptible to YCMDs because it contains repetitive sequence blocks (amplicons) organized into palindromes, which predispose the region to homologous intrachromosomal recombination (5,6). Clinically, these microdeletions are subdivided into three non-overlapping sub-regions: The proximal (AZFa), middle (AZFb) and distal (AZFc) segments (5,6).

Screening studies have indicated that the AZFc sub-region is the most frequently deleted locus (5,6,8-10). This deletion pattern is often associated with a higher probability of successful sperm recovery through testicular sperm extraction (TESE) and intracytoplasmic sperm injection (ICSI) (5,6). Conversely, deletions involving AZFa and AZFb, or their combinations (AZFb+c and AZFa+b+c) are associated with a near complete absence of testicular sperm; therefore, surgical retrieval is generally not recommended for these patients (6). Consequently, the molecular identification of AZF microdeletions has become a routine diagnostic requirement as recommended by the European Academy of Andrology (EAA) and European Molecular Genetics Quality Network (EMQN) (6).

Globally, YCMDs account for 10-15% of azoospermia cases and 5-10% of severe oligozoospermia (5,6,11). Nevertheless, the reported prevalence is highly heterogeneous, varying from 1.5 to 58.8% depending on ethnicity and geographic region (12,13). However, investigations on YCMDs in Libya remain limited. For instance, Teka *et al* (14) reported a prevalence of 3.3% in Misurata, Libya; however, their analysis relied on a limited set of single STS loci (sY81, sY164, and sY277), which are currently considered suboptimal owing to their repetitive sequences or polymorphic characteristics (15).

To ensure diagnostic accuracy, the current EAA/EMQN guidelines recommend multiplex PCR using a standardized set of STS primers: sY14 (SRY), ZFX/ZFY, sY84, sY86, sY127, sY134, sY254, and sY255. Therefore, the objective of the present study was to determine the prevalence and spectrum of AZF microdeletions in a sample of Libyan men with infertility using EAA/EMQN recommended standardized primers, thereby establishing a robust genetic baseline for this population.

## Patients and methods

### Study design and patient selection

This cross-sectional study included a cohort of 41 Libyan men with infertility, recruited by reproductive medicine specialists from three private fertility centers in Tripoli: The Nour Al-Hayat Fertility Clinic, Al Khalil Hospital and Yashfeen Clinic. The age of the participants ranged from 24 to 55 years, with a median age of 38 years. The cohort was stratified into two groups based on semen analysis profile: One group with azoospermia (n=23) and a non-azoospermia group (n=18). Detailed clinicopathological and hormonal characteristics of the participants, are presented in the Results section. Inclusion criteria were Libyan males presenting with primary infertility and non-obstructive spermatogenic impairment, including azoospermia, severe oligozoospermia, or qualitative defects such as oligoasthenoteratozoospermia (OAT). Conversely, patients with obstructive azoospermia, known chromosomal abnormalities (e.g., Klinefelter syndrome, 47, XXY), or infertility secondary to primary hormone imbalances were excluded. The initial clinical diagnostics were conducted at the recruitment clinics (Nour Al-Hayat, Al Khalil, and Yashfeen Clinic). Semen parameters were assessed manually following WHO 2010 standards to ensure inter-site consistency. Hormonal assays were performed using standardized Chemiluminescence Immunoassay (CLIA) platforms. The resulting data were then centralized and analyzed alongside the molecular results obtained at the Biotechnology Research Centre (BRC).

Participants were recruited and samples were collected over a 6-month period, from April, 2025 to October, 2025. Convenience sampling was adopted owing to the considerable cultural sensitivity surrounding male infertility in the region, which contributed to the high rate of non-participation. Ethical approval was obtained from the Bioethics Committee of the Biotechnology Research Centre (BRC) of Tripoli, Libya (Ref: NBC: 001.H.25.12). The study was conducted in strict accordance with the principles of the Declaration of Helsinki.

Informed consent (both written and verbal) was obtained from all participants regarding the use of their clinical and genetic data for research purposes. To ensure participant privacy, all personal identifiers were removed, and the data were fully anonymized prior to laboratory analysis. Data confidentiality was maintained throughout the study using a secure coded data collection system.

### Hormone and semen analyses

Serum levels of key reproductive hormones, including testosterone (T), follicle stimulating hormone (FSH), luteinizing hormone (LH) and prolactin (PRL), were quantified using electrochemiluminescence immunoassay (ECLIA) utilizing the cobas e 411 automated system; the products are supplied by Roche Diagnostics GmbH. The specific Elecsys^®^ reagent packs used were: Elecsys^®^ FSH: Ref. 08932352190, Elecsys^®^ LH: Ref. 07027575190; Elecsys^®^ Testosterone II: Ref. 08946353190; Elecsys^®^ Prolactin II: Ref. 03203093190. These assays were conducted following the manufacturer's standardized protocols at the affiliated clinical laboratories. Semen analysis and sperm parameters were evaluated in strict accordance with the WHO Laboratory Manual for the Examination and Processing of Human Semen, fifth edition (16). Semen samples were collected by masturbation into sterile containers after a period of 2 to 7 days of sexual abstinence. Following collection, samples were allowed to undergo complete liquefaction at 37˚C for 30 min. Sperm concentration, motility and morphology, were assessed using the Computer-Aided Semen Analysis (CASA) system. Following the confirmation of infertility through semen analysis and hormonal profiling at the participating clinics, patients were referred to the Biotechnology Research Center (BRC) for the molecular phase of the study. Peripheral blood samples (~3-5 ml) were collected (in the morning 9 to 11 a.m.) from each participant via venipuncture into sterile EDTA-containing tubes to prevent coagulation. Blood collection was performed by trained personal. The samples were immediately processed for genomic DNA extraction or stored at 4˚C (for a maximum of 24 h) or frozen at -20 to -80˚C until further analysis.

### YCMD analysis

Molecular screening for YCMDs was performed using the Sacace AZF System Y chromosome kit (REF 01200-50; ver. 12.12.2017; Sacace Biotechnologies). Genomic DNA was isolated from peripheral venous blood using the QIAamp DNA Blood Mini kit (Qiagen GmbH) according to the manufacturer's instructions. DNA samples were subsequently amplified using two multiplex fluorescent real-time PCR assays, following the best practice guidelines recommended by the EAA/EMQN (6). The Sacace system is designed to simultaneously target two STS loci within each AZF region across two separate multiplex master mixes: PCR Mix A which contains: sY86 (AZFa), sY127 (AZFb), and sY254 (AZFc), with internal controls ZFX/ZFY; PCR Mix B which contains: sY84 (AZFa), sY134 (AZFb), and sY255 (AZFc), with the sY14 (SRY) control. Amplification and detection were performed using the Bio-Rad CFX96 Real-Time PCR Detection System mounted on a C1000 Touch Thermal Cycler (Bio-Rad Laboratories, Inc.). Fluorescence signals were monitored across four channels: JOE/HEX/yellow for internal controls, FAM/green for AZFa, ROX/orange for AZFb and Cy5/red for AZFc. The thermal cycling conditions were strictly set according to the manufacturer's protocol. The thermocycling conditions consisted of an initial denaturation at 94˚C for 1 min 30 sec, followed by 40 cycles of denaturation at 94˚C for 15 sec, annealing at 64˚C for 40 sec, and extension at 72˚C. Fluorescence was acquired during the 64˚C step. Data interpretation was based on the presence or absence of characteristic sigmoidal (S-shaped) amplification curves. A positive amplification curve indicated the presence of the target STS (i.e., no deletion), whereas a flat baseline (i.e., absence of amplification) in the presence of a successful internal control signal indicated a microdeletion in the respective AZF region. All molecular procedures, including DNA extraction, quantification and qualitative PCR, were performed at the Genetic Engineering Unit of the BRC, Tripoli, Libya.

### Statistical analysis

Data were analyzed using descriptive and inferential statistical methods. Quantitative variables, including patient age and serum hormonal levels (FSH, LH, T and PRL), are expressed as the mean ± standard deviation (SD). Categorical variables, including the prevalence of YCMDs and semen phenotypes, are presented as frequencies and percentages. To account for the small sample size (n=41) and the low frequency of genetic events, 95% confidence intervals (CIs) for YCMD prevalence were calculated using the Clopper-Pearson exact binomial method. This approach was selected over the standard Wald method to provide a more conservative and reliable estimate of proportional uncertainty in a small clinical cohort. The independent-samples t-test was used to compare mean hormone levels between the azoospermia and non-azoospermia groups. Correlations between continuous variables (e.g. FSH vs. T and age vs. T) were evaluated using the Pearson's correlation coefficient (r) and visualized using scatter plots with linear regression fit lines. A P-value <0.05 was considered to indicate a statistically significant difference. All statistical analyses were performed using IBM SPSS Statistics for Windows, version 26.0 (IBM Corp.).

## Results

Semen analysis of the study cohort (n=41) revealed a heterogenous spectrum of spermatogenic impairments. Azoospermia was the most prevalent phenotype, observed in 56.1% (23/41) of the patients, followed by severe OAT 29.3% (12/41) and asthenoteratozoospermia 7.3% (3/41). Other categories, including severe oligozoospermia, teratozoospermia and moderate OAT, accounted for 2.4% (1/41) of the cohort. For comparative analysis, patients were stratified into two primary groups: One group with azoospermia (n=23) and a non-azoospermia group (n=18), enabling a robust evaluation of hormonal profiles and genetic prevalence.

**Figure 1:**
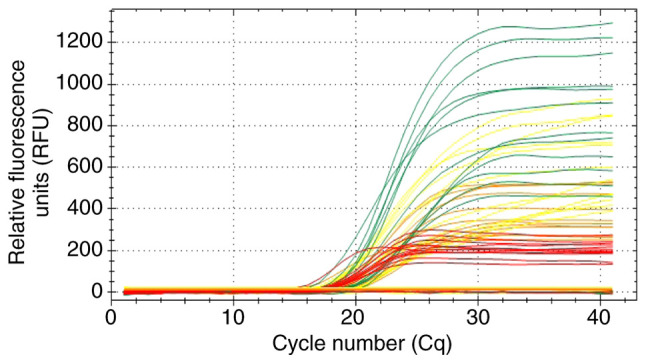
Representative real-time multiplex PCR amplification curves for AZF regions. Real-time-PCR plots illustrating the presence of intact genomic sequences for the azoospermia factor regions in 6 patients with infertility. Positive amplification is characterized by sigmoidal curves, indicating the presence of tested sequence tagged sites. Fluorescence channels represent specific target regions: JOE/HEX/yellow (internal control), FAM/green (AZFa), ROX/orange (AZFb), and Cy5/red (AZFc). The Y-axis represents relative fluorescence units (RFU) and the X-axis denotes the cycle number (Cq).

Genetic screening revealed a total YCMD prevalence of 2.44% (1/41). The azoospermia group, had a YCMD prevalence of 4.35% (1/23), whereas no deletions were detected in the non-azoospermia group. Owing to the small sample size, 95% CIs were estimated using the Clopper-Pearson exact method, yielding ranges of 0.06 to 12.79% for the total cohort and 0.11 to 21.95% for the azoospermia group (Table I). The remaining 97.56% (40/41) of the cohort, including 95.65% of patients with azoospermia, did not exhibit any detectable deletions, with all tested STSs for the AZFa, AZFb and AZFc regions exhibiting characteristic positive sigmoidal amplification curves (Fig. 1).

**Figure 2:**
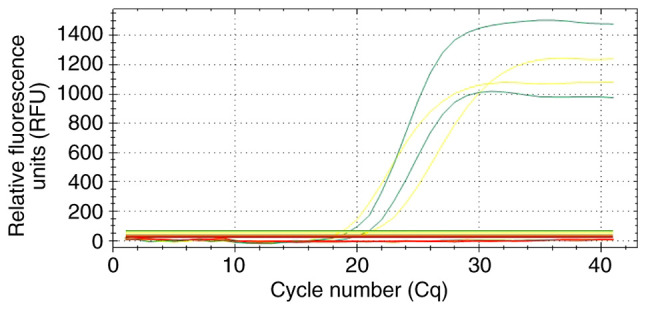
Real-time multiplex PCR profile illustrating a complete AZFb+c microdeletion. Amplification profile of a 29-year-old patient with azoospermia, identified with a complete deletion across the AZFb and AZFc subregions. While the JOE/HEX/yellow (internal control) and FAM/green (AZFa) channels show successful sigmoidal amplification, the flat baselines in the ROX/orange (AZFb) and Cy5/red (AZFc) channels confirm the absence of specific sequence tagged sites (sY127, sY134, sY254 and sY255). This molecular genetic profile is a hallmark of definitive spermatogenic failure. The Y-axis represents relative fluorescence units (RFU) and the X-axis denotes the cycle number (Cq).

A 29-year-old male patient with complete azoospermia and no family history of infertility tested positive for YCMD. Multiplex real-time PCR confirmed a complete microdeletion spanning the AZFb and AZFc sub-regions, as evidenced by the absence of amplification for the sY127, sY134, sY254 and sY255 loci (Fig. 2).

This genetic profile corresponded to a hypergonadotropic phenotype, characterized by elevated FSH levels (11.39 mIU/ml) and high-normal LH levels (7.78 mIU/ml; Table II), indicating substantial spermatogenic failure. Notably, the FSH level of the patient was lower than the levels of the azoospermia group mean of 18.04 mIU/ml (Table III), illustrating the hormonal variability inherent in non-obstructive azoospermia (NOA). The levels of T (5.27 ng/ml) and PRL (8.85 ng/ml) remained within their respective normal reference ranges, which is consistent with compensated primary testicular failure. Despite a normal ejaculate volume (3.1 ml), pH (8) and liquefaction time (Table II), complete azoospermia was confirmed (0.00 million/ml).

The identification of a complete AZFb+c deletion in this patient serves as a definitive negative prognostic indicator. According to the EAA and EMQN guidelines, such deletions are diagnostic of the complete absence of germ cells (Sertoli cell-only syndrome) or complete spermatogenic maturation arrest. Consequently, surgical interventions, such as TESE or micro-TESE, are contraindicated, as the probability of retrieving viable spermatozoa is negligible. Such a diagnosis enables an immediate transition to alternative reproductive counselling, such as sperm donation or adoption, thereby sparing the patient unnecessary invasive surgical procedures.

**Figure 3:**
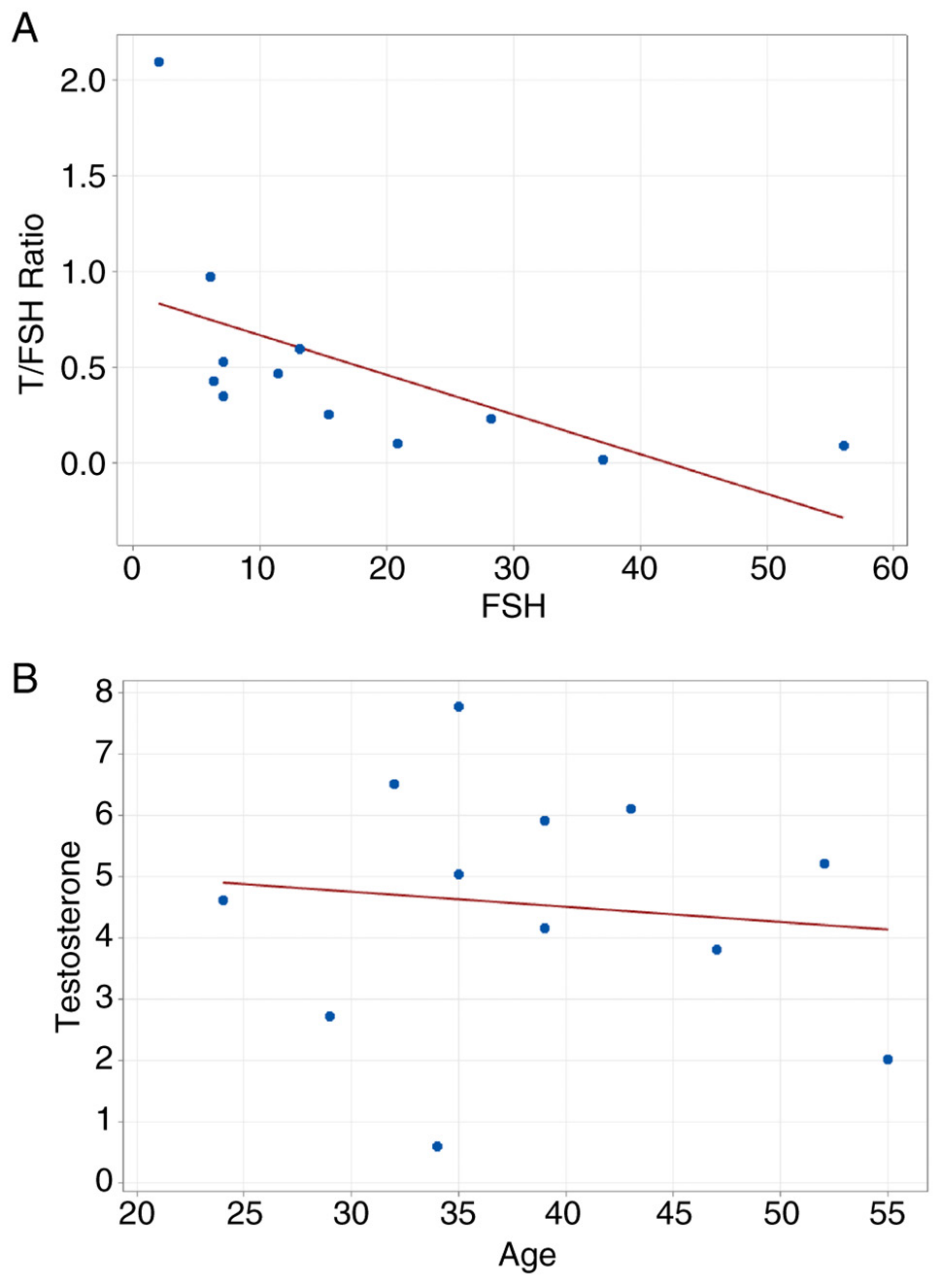
Scatter plots representing Pearson's correlation analysis of hormonal and clinical parameters. (A) Scatter plot displaying the moderate negative correlation (r=-0.56) between the T/FSH ratio and FSH levels; which supports the study finding that higher FSH levels are associated with a significant decline in the T/FSH ratio, characteristic of primary testicular failure. (B) Scatter plot displaying the negligible negative correlation (r=-0.12) between patient age and testosterone levels. These scatter plots confirm that hormonal imbalances in this cohort were driven by spermatogenic status rather than age. Solid lines represent the linear regression fit. FSH, follicle stimulating hormone; T, testosterone.

Comparative analysis of clinical and hormonal parameters between the azoospermia and non-azoospermia groups revealed significant differences, as summarized in the clinicopathological data presented in Table III. The mean FSH level in the azoospermia group (18.04±14.62 mIU/ml) was ~4-fold higher than that in the non-azoospermia group (4.75±2.45 mIU/ml; P<0.001). The azoospermia group exhibited significantly higher LH levels (10.96±7.67 mIU/ml) than the non-azoospermia group (4.84±2.27 mIU/ml; P<0.01). The LH/FSH ratio was suppressed in the azoospermia group compared with the non-azoospermia group (0.61 vs. 1.19; P<0.01), supporting a primary testicular etiology. The T/LH ratio was significantly lower in the azoospermia group (-0.37 vs. 0.87; P<0.001), suggesting that Leydig cells require higher LH stimulation to maintain normal testosterone levels. Within the azoospermia group, the T/FSH ratio exhibited a moderate negative correlation (r=-0.56), supporting the diagnosis of primary testicular failure (Fig. 3A). A negligible correlation was observed between age and testosterone (r=-0.12, Fig. 3B), suggesting that these hormonal imbalances are more likely attributable to an underlying pathology rather than natural ageing.

Additionally, the azoospermia group exhibited a high degree of variability in FSH levels (18.04±14.62 mIU/ml), as reflected by an elevated coefficient of variation. This substantial variance (relative to the mean) underscores the clinical heterogeneity of the group, which likely encompasses a spectrum of histopathological states ranging from Sertoli cell-only syndrome (characterized by elevated FSH values) to spermatogenic maturation arrest (associated with moderate FSH elevation).

## Discussion

According to the EAA/EMQN guidelines, multiplex PCR remains the gold standard method for the detection of YCMDs (6). This method provides a resolution (0.8-4.0 Mb) that exceeds that of traditional G-banding cytogenetics, which is limited to detecting deletions >5-10 Mb (3,4). The present study utilized multiplex fluorescent RT-PCR to investigate AZF microdeletions in 41 Libyan men with infertility, utilizing two STS loci per AZF region (sY84 and sY86 for AZFa; sY127 and sY134 for AZFb; and sY254 and sY255 for AZFc) alongside ZFX/ZFY and SRY controls to ensure diagnostic rigor. The results revealed a complete absence of amplification for the sY127, sY134, sY254 and sY255 loci (Fig. 2), which signifies a physical genomic deletion corresponding to AZFb+c microdeletion. The technical validity of the detected AZFb+c microdeletion is supported by the adherence to the EAA/EMQN best practice guidelines and the manufacturer's validated diagnostic protocol. Specifically, the identification of a microdeletion is based on the total absence of target-specific amplification (flat baseline) in the presence of a confirmed internal control signal (SRY and ZFX/ZFY), indicating a complete physical absence of the genomic segment (i.e., there is no DNA template to amplify) in the DNA of the patient. As a microdeletion represents a nullisomic state for the targeted loci, there is no PCR product to sequence. Sequencing is typically employed to identify point mutations, polymorphisms, or small indels within an existing DNA sequence, whereas the diagnosis of classic AZF deletions characterized by large-scale genomic losses relies fundamentally on the confirmed absence of STS amplification, rendering the findings of the present study definitive within the established diagnostic framework.

The present study identified a single deletion exclusively within the azoospermia group (1/23; 4.35%, Table I and Fig. 2), consistent with reports that such deletions are markedly more prevalent in men with azoospermia than in those with severe oligozoospermia (5,6,8,17). Additionally, the total prevalence of YCMDs (2.44%; 1/41, 95% CI, 0.06-12.79%) in the present study cohort is indicative rather than definitive of the broader infertile Libyan male population, given the limitations of the study, including the small sample size and the absence of a normozoospermic control group. However, the clinical validity of the detected YCMDs remains robust, as complete AZF deletions are virtually absent in fertile populations globally (5,6,10) and regionally (14,18). Larger multicenter studies involving both infertile and control populations are required to accurately determine the true prevalence in Libya.

Nonetheless, the prevalence rate of 2.44% was consistent with the lower end of the global range and with findings from regional studies. For example, a YCMD prevalence of 2% was previously reported in Germany and Austria (5); a large multi-ethnic cohort in London reported a prevalence of 4% (19), and a study of 1,030 Japanese men with infertility reported a prevalence of ~7% (20). This finding is also consistent with regional studies (Table IV), including a previous study in Misurata, Libya, which reported a 3.3% deletion rate (14), and an unpublished study in Tripoli, which reported a prevalence of 2.5% (A. H. Elnfati, personal communication, March 10, 2025). These figures align closely with data from Algeria (2%) (21), Tunisia (1.9-9.5%) (22,23), Morocco (3-9%) (24,25), the United Arab Emirates (2%) (26), Saudi Arabia (3.1%) (27) and Lebanon (2.5%) (28). By contrast, the prevalence rate was notably lower than that reported in Sudan (58.8%) (13), Iraq (47.8%) (29) and Egypt (16%) (30). Such extensive variations in prevalence are likely attributable to geographical differences, ethnicity, patient selection criteria, and specific STS markers (5,6). Additionally, high rates of consanguinity in certain regions or exposure to specific environmental toxins may contribute to an increased genetic susceptibility to YCMDs.

The identified AZFb+c deletion is of considerable clinical relevance, serving as both a diagnostic indicator of genetic infertility and a definitive negative prognostic indicator. Indeed, the specific type of AZF deletion dictates prognosis. According to the EAA/EMQN guidelines, the probability of retrieving viable spermatozoa via TESE/micro-TESE is negligible in cases of AZFa, AZFb and AZFb+c deletions. By contrast, AZFc deletions are associated with a 50-80% likelihood of sperm retrieval, although those carry a documented risk of vertical transmission to male offspring following TESE/ICSI (5,6,8,31,32), for which genetic counselling is mandatory. The detection of the AZFb+c genetic profile underscores the critical role of genetic screening in preventing unnecessary or unsuccessful surgical interventions, even in smaller clinical settings and facilitates an immediate transition to alternative counselling (e.g., sperm donation or adoption).

The low prevalence of YCMDs in the present study cohort suggests that other factors, such as autosomal recessive mutations, in *CEP78*, *SPAG17* and *TENT5D* (33-35), may be responsible for male infertility in Libya, potentially driven by high rates of regional consanguinity. These mutations disrupt sperm head development and flagellar formation, leading to a severe OAT phenotype (36,37). The present study cohort was characterized by severe quantitative (azoospermia) and qualitative (OAT) sperm abnormalities, despite the identification of only a single AZFb+c deletion. Therefore, future studies in Libya are required to incorporate whole-exome-sequencing approaches to identify autosomal recessive pathogenic variants.

Semen analysis within the present study cohort revealed a predominance of azoospermia (56.1%) and severe OAT (29.3%). These findings differ from earlier studies conducted in Western Libya, in which asthenozoospermia was often more prevalent (14,38,39). Across the Middle East and North African region, considerable phenotypic variation has been reported; for instance, azoospermia predominated in Algeria (61.3%) (21), whereas higher rates of asthenozoospermia have been documented in Iraq (40) and Sudan (41). Despite these variations, a common finding is that azoospermia and OAT frequently represent primary phenotypes among Arab men seeking fertility treatment (42,43).

Comparative hormonal data of the present study cohort revealed markedly elevated FSH levels in patients with azoospermia, hallmark feature of NOA. This elevation reflects the loss of negative feedback inhibition by inhibin B secondary to germ cell depletion (44). YCMDs are predominately observed in men with NOA (5,6,8), supporting the established genotype phenotype correlation (i.e., AZFb+c deletion/high FSH). The high coefficient of variation in FSH levels in the azoospermia group (18.04±14.62) reflects the clinical heterogeneity of the cohort, encompassing a spectrum from maturation arrest to complete germ cell absence.

Furthermore, the LH/FSH ratio (0.61) and the reduced T/LH ratio (0.37) in patients with azoospermia reflect functional testicular failure and impaired Leydig cell function. Additionally, the T/FSH ratio demonstrated greater sensitivity as an indicator of complete spermatogenic arrest in azoospermia (r=-0.56) compared with its association with partial sperm count variation in the non-azoospermia group (r=-0.27). These ratios provide greater diagnostic utility in NOA than isolated hormone levels (45). Given that >95% of patients with azoospermia are negative for YCMDs, these hormonal markers remain the primary tools for diagnosing functional testicular failure in idiopathic cases.

In conclusion, the detection of YCMDs within the AZF region represents a key genetic investigation in the diagnostic evaluation of male infertility. Screening for AZF microdeletions is essential for the accurate genetic diagnosis of NOA and for guiding personalized reproductive management. Therefore, studies on the prevalence, types and characteristics of YCMDs are of critical importance in reproductive medicine and fertility clinics. To the best of our knowledge, the present study provides the first genetic and phenotypic characterization of male infertility in Tripoli, Libya using EAA/EMQN standards. The identified AZFb+c deletion serves as a definitive negative prognostic indicator, rendering surgical sperm retrieval a contraindicated. Although the identified genetic prevalence was relatively low (2.44%), the distinct hormonal signature characterized by hypergonadotropism and reduced steroidogenic efficiency provides a robust clinical baseline for diagnosing primary testicular failure within this population. The high frequency of severe phenotypes (azoospermia and OAT) in the absence of YCMDs suggests that autosomal recessive mutations, potentially driven by regional consanguinity or environmental factors, may substantially contribute to male infertility in Libya. Routine AZF screening in Libyan fertility clinics is recommended to optimize clinical management and prevent unnecessary invasive procedures. The primary limitation of the present study is the relatively small sample size, which may not fully represent the true prevalence and spectrum of YCMDs across the Libyan population. Consequently, further large-scale screening studies incorporating larger sample sizes of patients with control cases and broader geographic representation are warranted to obtain a more accurate and comprehensive genetic landscape of male infertility in Libya.

## Figures and Tables

**Table I:** Prevalence and confidence intervals of YCMDs in the present study cohort.

Patient group	No. of patients	YCMDs	Prevalence (%)	95% CI (exact)
Azoospermia	23	1	4.35	0.11-21.95
Non-azoospermia	18	0	0.00	0.00-18.53
Total cohort	41	1	2.44	0.06-12.7s

YCMDs, Y chromosome microdeletions; CI, confidence interval.

**Table II:** Clinical and hormonal profile of the patient with AZFb+c microdeletion.

Parameter	Patient value	Reference range	Clinical interpretation
Hormone profile			
FSH	11.39 mIU/ml	1.5-8.0 mIU/ml	Elevated (spermatogenic failure)
LH	7.78 mIU/ml	1.8-8.0 mIU/ml	High-normal (compensated)
Testosterone	5.27 ng/ml	2.8-8 ng/ml	Normal
Prolactin	8.85 ng/ml	7.0-20.0 ng/ml	Normal
Semen analysis			
Volume	3.1 ml	≥1.5 ml	Normal
pH	8	7.2-8.6	Normal
Liquification	<20 min	<60 min	Normal
Sperm count	0.00 million/ml	≥39 million/ml	Azoospermia

AZF, azoospermia factor; FSH, follicle stimulating hormone; LH, luteinizing hormone.

**Table III:** Clinicopathological data categorized by semen analysis profile: Azoospermia and non-azoospermia.

	Clinical parameter (reference range)								
Patient group	Age (years)	Sperm count million/ml	FSH (1.5-12.4 mIU/ml)	LH (1.2-8.6 mIU/ml)	T (3.0-10.0 ng/ml)	PRL (2.0-18.0) ng/ml	LH/FSH ratio (r)	T/FSH ratio (r)	T/LH ratio (r)
Azoospermia (n=23)	37.3±6.9	0.00	18.04±14.62	10.96±7.67	4.08±1.90	13.34±5.86	0.61	-0.56	0.37
Non-azoospermia (n=18)	40.8±7.5	14.1±86	4.75±2.45	4.84±2.27	4.23±1.76	13.25±8.21	1.19	-0.27	0.87
P-value	P>0.05	P<0.001	P<0.001	P<0.01	P>0.05	P>0.05	P<0.01	P<0.01	P<0.001

FSH, follicle-stimulating hormone; LH, luteinizing hormone; T, testosterone; PRL, prolactin. P<0.05 was considered to indicate a statistically significant difference. Non-azoospermia (includes severe oligozoospermia, severe oligoasthenoteratozoospermia, oligoasthenoteratozoospermia asthenoteratozoospermia, and teratozoospermia). Values for hormone ratios are expressed as the Pearson's correlation coefficient (r).

**Table IV:** Prevalence of YCMDs in the AZF region across selected countries.

Country	Prevalence (%)	AZF deletion type	(Refs.)
Tripoli, Libya	2.44	AZFb+c	Present study
Misurata, Libya	3.3	AZFc, AZFb+c	(14)
Tripoli, Libya	2.5	AZFc	(A.H. Elnfati, personal communication, March 10, 2025)
Algeria	2.0	AZFc	(21)
Tunisia	1.9-9.5	AZFb, AZFc, AZFb+c	(22,23)
United Arab Emirates	2.0	AZFc	(26)
Lebanon	2.5	AZFc, AZFb+c	(28)
Saudi Arabia	3.1	AZFc	(27)
Morocco	3.0-9.0	AZFa, AZFb, AZFc, AZFa+b, AZFa+c, AZFb+c	(24,25)
Egypt	16.0	AZFb, AZFc, AZFb+c	(30)
Iraq	47.8	AZFb, AZFc, AZFb+c	(29)
Sudan	58.8	All types (AZFa was the most common)	(13)
Germany and Austria	2	-	(5)
London, UK	4	AZFa, AZFb, AZFc, AZFb+c	(19)
Japan	7	AZFa, AZFb, AZFc, AZFb+c	(20)

YCMDs, Y chromosome microdeletions; AZF, azoospermia factor.

## Data Availability

The data generated in the present study may be requested from the corresponding author.
